# Inhibition of the PIN1-NRF2/GPX4 axis imparts sensitivity to cisplatin in cervical cancer cells: PIN1/NRF2/GPX4 axis in CC

**DOI:** 10.3724/abbs.2022109

**Published:** 2022-08-17

**Authors:** Zheng Zhang, Qiangsheng Hu, Shuang Ye, Libing Xiang

**Affiliations:** 1 Ovarian Cancer Program Department of Gynaecologic Oncology Zhongshan Hospital Fudan University Shanghai 200031 China; 2 Department of Thoracic Surgery Shanghai Pulmonary Hospital Tongji University School of Medicine Shanghai 200433 China; 3 Department of Gynecologic Oncology Fudan University Shanghai Cancer Center Shanghai 200032 China; Department of Oncology Shanghai Medical College Fudan University Shanghai 200031 China

**Keywords:** cervical cancer, ferroptosis, chemoresistance, PIN1, GPX4

## Abstract

The incidence of cervical cancer (CC) ranks the fourth in female malignant tumors globally. Chemoresistance is one of the main causes of treatment failure in advanced recurrent CC. Prolyl isomerase 1 (PIN1) is overexpressed in a variety of tumors, and is closely associated with the malignant potential of tumor cells, such as transformation, proliferation, invasion and metastasis. In the present study, we demonstrate that cell death induced by suppression of PIN1 could be inhibited by ferrostatin-1 (Fer-1) and ferroptosis biomarkers including lactate dehydrogenase (LDH) release, lipid peroxidation and malondialdehyde (MDA) are upregulated by downregulating PIN1. We then discover that abrogation of PIN1 greatly decreases the level of glutathione peroxidase 4 (GPX4) and the level of PIN1 is positively correlated with the level of GPX4. Furthermore, the knockdown of PIN1 promotes ferroptosis induced by RSL3. The mechanism involves PIN1 silencing which downregulates GPX4 by decreasing the level of nuclear factor E2-related factor 2 (NRF2). Furthermore, overexpression of NRF2 inhibits RSL3-mediated ferroptosis of CC cells when PIN1 is silenced. In addition, our results indicate that cisplatin (DDP) induces ferroptosis, which is restrained by overexpression of PIN1. The PIN1 inhibitor, KPT-6566, promotes the cytotoxic effect of DDP. The present study reveals that PIN1 affects ferroptosis and sensitivity to DDP in CC cells via the NRF2/GPX4 axis, thereby identifying PIN1 as a potential therapeutic target for CC.

## Introduction

Cervical cancer (CC) is the fourth most common cancer in women, with 342,000 deaths per year globally [ 1, 2] . Radical surgery often has a satisfactory prognosis for patients with early-stage CC [ 3, 4] . Chemotherapy based on cisplatin (DDP) is a key treatment strategy for metastatic or recurrent CC and concurrent chemoradiotherapy can significantly improve the prognosis of patients with advanced-stage CC [5]. However, chemoresistance often causes therapy failure and resistance to DDP also results in tumor recurrence and a low survival rate [6]. To date, the exact mechanism of DDP resistance remains unclear. Therefore, determination of the molecular mechanism of chemoresistance and improving the chemosensitivity of CC are urgently required for the comprehensive treatment of CC.

In general, DDP can induce DNA damage and prevent cells entering the G1 phase, apoptosis, autophagy and so on [7]. However, activation of intracellular anti-apoptosis and anti-autophagy may mediate apoptosis escape and then chemotherapy resistance [8]. Thus, exploring other non-apoptotic forms of death induced by chemotherapy to eliminate tumor cells and control the expansion of chemotherapy-resistant clones could provide a novel treatment strategy to improve the poor prognosis of CC patients. Ferroptosis is an iron-dependent and non-apoptotic form of cell death characterized by the accumulation of intracellular reactive oxygen species (ROS) [9]. Ferroptosis is closely correlated with the occurrence and progression of many diseases such as metabolic and neurodegenerative diseases and tumors [ 10– 12] . Several studies have reported that DDP resistance may be regulated by ferroptosis, which can synergistically kill cancer cells together with DDP [ 13, 14] .

Prolyl isomerase 1 (PIN1) is a member of the microcin protein subfamily of peptidyl-prolyl cis/trans isomerase, which specifically binds to the pSer/Thr motif of the substrate protein and catalyzes the isomerization of its peptide bond [15]. PIN1 mediates the function, activity and cellular localization of target proteins by altering their structures [ 15, 16] . Therefore, PIN1 is involved in the mediation of cell cycle, gene transcription and signal transduction [ 17, 18] . Previous studies have shown that PIN1 dysfunction is closely related to the occurrence of tumors [ 19, 20] . Li *et al*. [21] confirmed that PIN1 could increase the level of cyclin D1 and promote the tumorigenesis of CC. Ma *et al*. [22] reported that PIN1 could regulate the epithelial-mesenchymal transition and then affect the invasion and metastasis of CC cells (CCCs). Guo *et al*. [23] demonstrated that a new PIN1 inhibitor, KPT-6566, inhibited CCCs. It is suggested that PIN1 could be used as an oncogene to regulate the occurrence, development, invasion and metastasis of CC. However, the relationship between PIN1 and ferroptosis has not been reported.

In the present study, we analyzed the effect of PIN1 on ferroptosis and its potential mechanism in CCCs. We found that abrogation of PIN1 promoted ferroptosis and increased the sensitivity of CCCs to DDP, which was partly due to suppression of the NRF2/GPX4 axis. Our findings revealed that PIN1/NRF2/GPX4 might be a new treatment target with the potential for improving the chemotherapy effect in CC.

## Materials and Methods

### Cell lines and small compounds

The cervical carcinoma cell lines, ME-180 and SiHa, were obtained from the American Type Culture Collection (ATCC; Manassas, USA) and the culture conditions were consistent with the ATCC protocol. Thus, the SiHa and ME-180 cells were cultured in Dulbecco’s modified Eagle’s medium (Gibco, Carlsbad, USA) containing 10% fetal bovine serum (Gibco), 100 U/mL penicillin (Gibco) and 0.1 μg/mL streptomycin (Gibco). The cells were cultured in incubators with 5% CO _2_ at 37°C. RSL3 (HY-100218A), 3-methyladenine (HY-19312), Z-VAD-FMK (HY-16658B), ferrostatin-1 (HY-100579) and DDP (HY-17394) were purchased from MedChemExpress (Monmouth Junction, USA).

### Quantitative real-time PCR (qRT-PCR)

Trizol reagent (Invitrogen, Carlsbad, USA) was used to isolate and extract total RNA. Reverse transcription was then conducted using a PrimeScript RT reagent kit (TaKaRa, Dalian, China) to obtain cDNA. The diluted cDNA and 5.6 μL of reaction solution were configured into a 10 μL reaction system, which was then added to the 384-well plate for detection. The reaction conditions were set as follows: pre-denaturation at 95°C for 30 s; denaturation at 95°C for 15 s; annealing and extension at 60°C for 30 s. The reaction went through 42 cycles, and finally, the melt curve was drawn. The levels of genes were detected using an ABI 7900HT Real-time PCR system (Applied Biosystems, Foster City, USA) and gene expression was calculated using the 2 ^−ΔΔCT^ method. *β*- *Actin* was used as the internal control. The primers used are listed in Table 1.

**Table:** **Table 1** Sequences of primers used in this study

Gene	Sequence (5′→3′)
*β-actin* forward	CTACGTCGCCCTGGACTTCGAGC
*β-actin* reverse	GATGGAGCCGCCGATCCACACGG
*PIN1* forward	GAGAAGATCACCCGGACCAAGGAG
*PIN1* reverse	TCCGCAGCGCAAACGAGGCGTCTTC
*SLC7A11* forward	GCTGTGATATCCCTGGCATT
*SLC7A11* reverse	GGCGTCTTTAAAGTTCTGCG
*ACSL4* forward	CTGTTCAGCGTTTTGCAAGGTA
*ACSL4* reverse	TAGTGGCATCTCCCTGGTCC
*GPX4* forward	ACCGAAGTAAACTACACTCAG
*GPX4* reverse	GGCGAACTCTTTGATCTCTT
*NRF2* forward	CACATCCAGTCAGAAACCAGTGG
*NRF2* reverse	GGAATGTCTGCGCCAAAAGCTG

### Western blot analysis

Western blot analysis was performed as described previously [24]. The antibodies used in this study were as follows: β-actin (1:4000; Proteintech, Chicago, USA), PIN1 (1:1000; Proteintech), NRF2 (1:1000; Abcam, Cambridge, UK), ACSL4 (1:1000; Proteintech), SLC7A11 (1:1000; CST, Beverly, USA) and GPX4 (1:1000; Proteintech), HRP-conjugated secondary antibodies (1:3000; Proteintech).

### Plasmids

The short hairpin RNA (shRNA) constructs against PIN1 were generated using the pLKO.1 TRC cloning vector (Addgene plasmid 10878; Cambridge, USA). The sequences (21 bp) against PIN1 were 5′-GCCATTTGAAGACGCCTCGTT-3′ (sense) and 5′-AGGAGAAGATCACCCGGACCA-3′ (antisense) [25]. pLKO.1-sh-scramble (Addgene plasmid 1864) was used as a control plasmid. Flag-tagged PIN1 and NRF2 were cloned into a pCDH-CMV-MCS-EF vector (System Biosciences, Carlsbad, USA), which were used to generate stable PIN1- and NRF2-overexpressing cells.

### Cell viability

Cell viability was determined using the Cell Counting Kit-8 kit (CCK-8;Dojindo Laboratories, Tokyo, Japan) following the manufacturer’s instructions. In brief, 4000 cells per well were seeded in 96-well plates, 10 μL CCK-8 reagent was added to each well and the plates were incubated in the cell incubator for 1 h. The absorbance of each well was detected at 450 nm utilizing a microplate reader (Thermo Fisher Scientific, Waltham, USA).

### Colony formation assay

The cells were seeded and cultivated in 6-well plates at 500 cells per well for 10 days. Afterwards, the cells were fixed using 4% paraformaldehyde and then stained with 0.1% crystal violet. The colonies were then counted under a microscope (Olympus, Tokyo, Japan).

### Lipid peroxidation assay

BODIPY™ 581/591 C11 (D3861; Thermo Fisher Scientific) was used to determine lipid peroxidation. Briefly, after treatment with the test agents for the indicated time, cells were obtained by trypsinization. Next, the cells were resuspended in a basic medium containing BODIPY™ 581/591 C11 (2 μM). The cells were then incubated for 30 min in a cell incubator. Finally, these samples were detected and analyzed using a flow cytometer (BD, Monmouth Junction, USA) and data were obtained from the FL1 channel. In addition, adherent cells were directly incubated with the reagent (2 μM) for 30 min in a cell incubator. Fluorescence was then detected using a confocal fluorescence microscope (Olympus).

### MDA assay

The level of malondialdehyde (MDA) was analyzed using a Lipid Peroxidation MDA Assay kit (S0131M; Beyotime, Shanghai, China) following the manufacturer’s protocol. In brief, the lysed cells were centrifuged at 12,000 *g* for 10 min to obtain the supernatant. MDA reagent was then fully mixed with supernatant. The mixture was heated at 100°C for 15 min and then cooled to room temperature in a water bath. The mixture was centrifuged at 1000 *g* at room temperature for 10 min. Then, 200 μL supernatant was added to each well of the 96-well plate, and then the absorbance was measured at 532 nm with a microplate reader (Thermo Fisher Scientific).

### LDH release assay

A lactate dehydrogenase (LDH) kit (Nanjing Jiancheng Bioengineering Institute, Nanjing, China) was used to detect the level of LDH release. CCCs were cultured in 6-well plates for 48 h. The supernatant was subsequently obtained and the CCCs were treated with 1.5% Triton X-100. The supernatant and lysed samples were then cultured with 2,4-dinitrophenylhydrazine and coenzyme I for 15 min. The absorbance value was obtained at 490 nm.

### Immunohistochemical staining (IHC)

Human CC specimens were obtained from patients in Fudan University Shanghai Cancer Center, with patients’ consent and approval from the Institutional Research Ethics Committee. IHC was performed using antibodies against PIN1 and GPX4 following standard procedures. Anti-PIN1 (10495-1-AP; Proteintech) and anti-GPX4 (67763-1-Ig; Proteintech) were utilized at dilutions of 1:100 and 1:500, respectively. The intensity and positive proportion were semi-quantitatively scored as previously described [26].

### Statistical analysis

Statistical analyses were conducted using the GraphPad Prism 7. Data are expressed as the mean±SD. *P*<0.05 was considered to be statistically significant

## Results

### Cell death induced by inhibition of PIN1 can be suppressed by ferroptosis inhibitor

**Figure 1:**
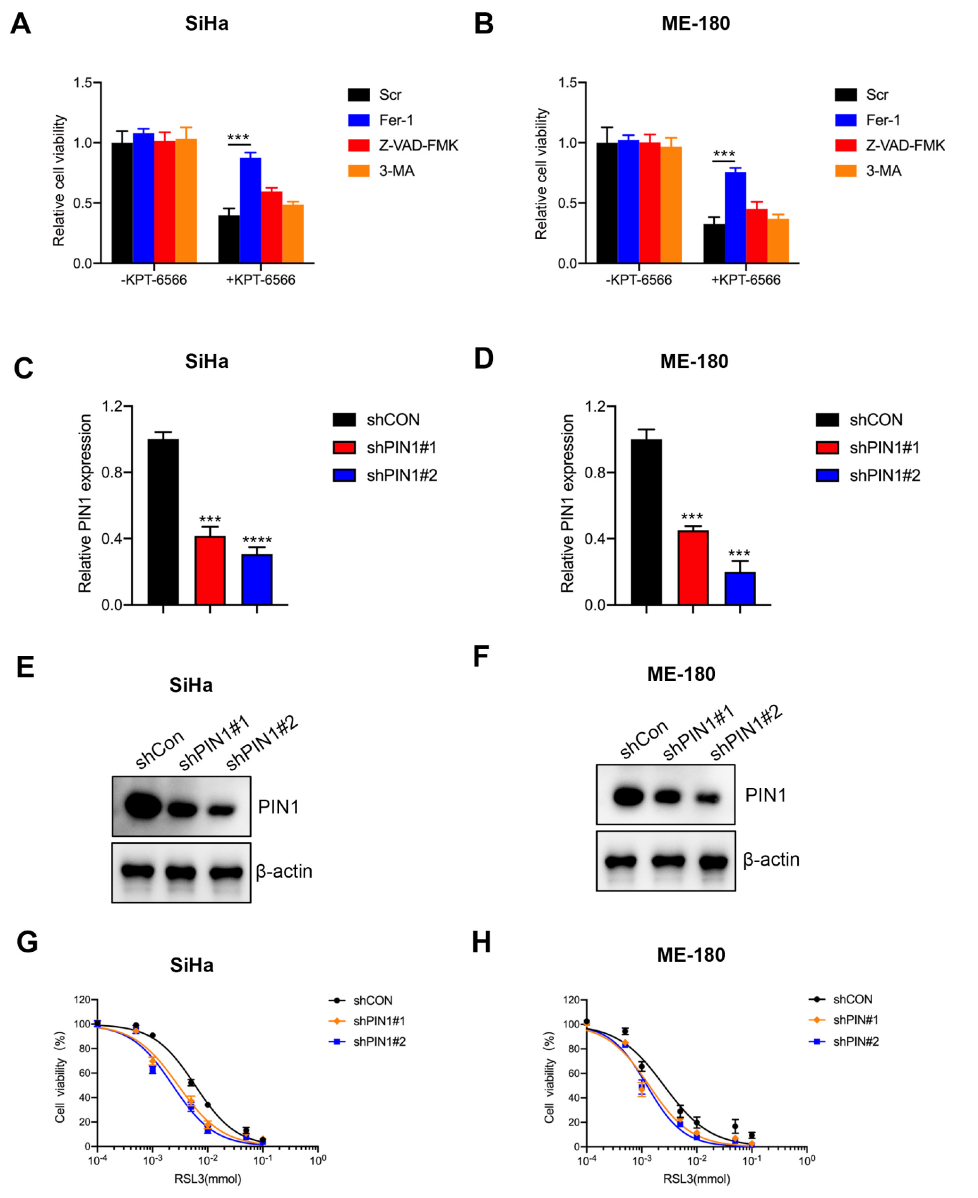
Figure 1 Cell death induced by inhibition of PIN1 is suppressed by the ferroptosis inhibitor (A,B) CCK-8 was used to detect the viability of SiHa and ME-180 cells treated with KPT-6566, Fer-1, Z-VAD-FMK and 3-MA, respectively. (C,D) qRT-PCR was used to evaluate the knockdown efficiency of shPIN1 in SiHa and ME-180 cells. (E,F) Western blot analysis was used to evaluate the knockdown efficiency of shPIN1 in SiHa and ME-180 cells. (G,H) CCK-8 was used to analyze the viability of PIN1-silenced SiHa and ME-180 cells treated with RSL3. *** P<0.001, and **** P<0.0001.

To investigate the role of PIN1 in ferroptosis of CCCs, we assessed cell viability in the human CC cell lines SiHa and ME-180. Firstly, the PIN1 inhibitor, KPT-6566, promoted cell death and this effect was inhibited by Fer-1, while autophagy inhibitor, 3-Methyladenine (3-MA), or apoptotic inhibitor, Z-VAD-FMK, failed to reverse cell death induced by the inhibition of PIN1 ( Figure 1A,B). Subsequently, stable PIN1-silenced SiHa and ME-180 cells were established by infection with lentivirus and selected using puromycin. The knockdown efficiency was verified by qRT-PCR ( Figure 1C,D) and western blot analysis ( Figure 1E,F). To determine whether PIN1 could mediate ferroptosis in CCCs, we silenced PIN1 and synchronously treated the cells with the ferroptosis inducer, RSL3. The results showed that the abrogation of PIN1 enhanced RSL3-induced ferroptosis ( Figure 1G,H). These results indicated that cell death mediated by knockdown of PIN1 was partly through ferroptosis.

### Knockdown of PIN1 causes lipid peroxidation in CCCs

**Figure 2:**
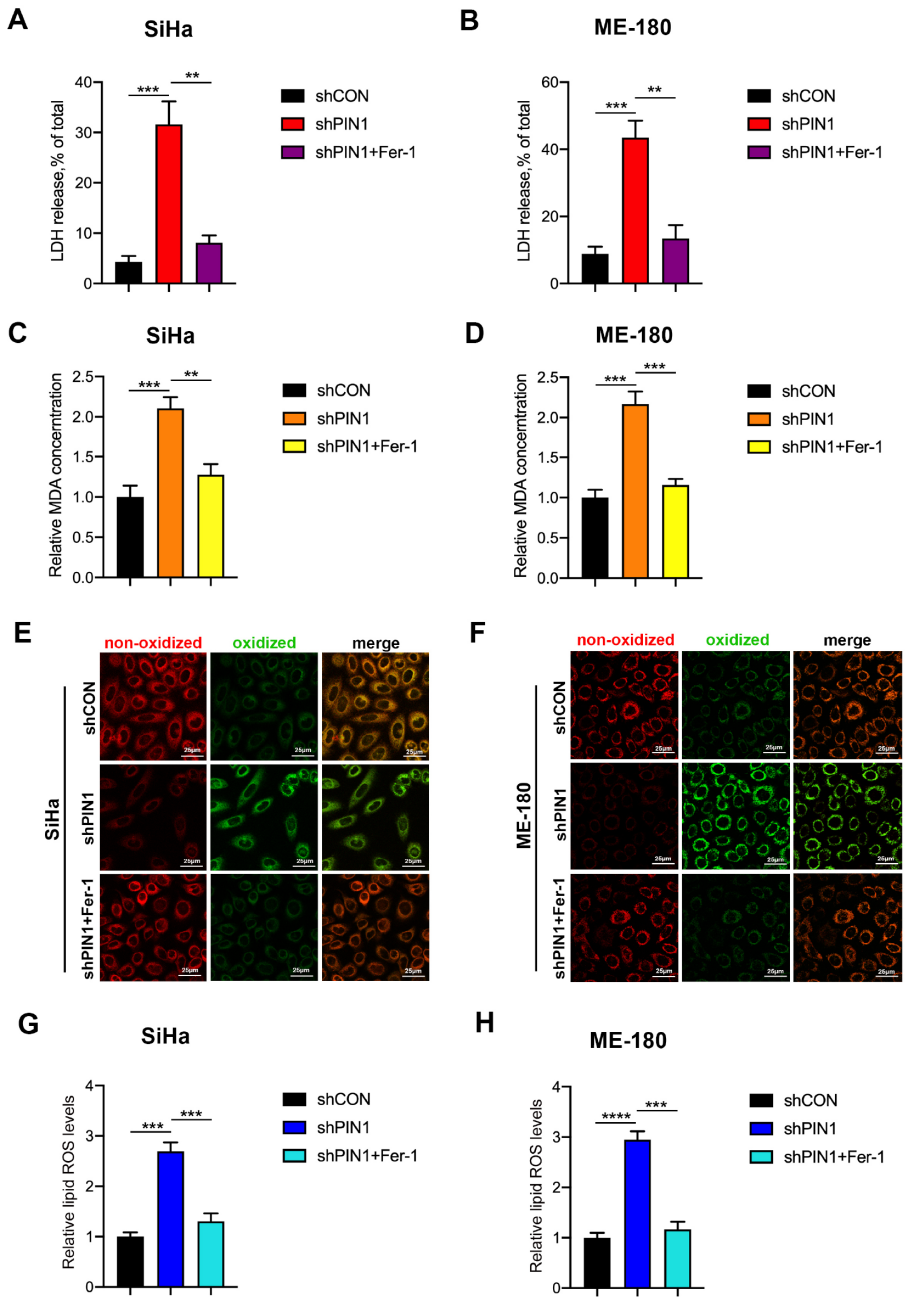
Figure 2 Knockdown of PIN1 causes lipid peroxidation in CCCs (A,B) LDH release was determined in PIN1-silenced SiHa and ME-180 cells treated with Fer-1. (C,D) The levels of MDA were analyzed in PIN1-silenced SiHa and ME-180 cells treated with Fer-1. (E,F) The oxidized lipid in PIN1-silenced SiHa and ME-180 cells treated with Fer-1 was analyzed using confocal microscopy. Scale bar=25 μm (G,H) The lipid ROS in PIN1-silenced SiHa and ME-180 cells treated with Fer-1 was analyzed using flow cytometry. ** P<0.01, *** P<0.001, and **** P<0.0001.

Ferroptosis can induce lethal damage and perforation of the lipid membrane. Thus, we assessed LDH release. As expected, silencing PIN1 significantly upregulated the level of released LDH, which was inhibited by Fer-1 ( Figure 2A,B). Additionally, inhibition of PIN1 increased MDA, which was also suppressed by Fer-1 ( Figure 2C,D). We then used BODIPY™ 581/591 C11, a fluorescent probe, to directly determine oxidized lipids. When the probe binds to oxidized lipids, the fluorescence changes from red to green, which can be detected by flow cytometry. The results indicated that downregulation of PIN1 increased oxidized lipids and the effect was reversed by Fer-1 ( Figure 2E,F). Similarly, inhibition of PIN1 greatly increased lipid ROS, which were decreased by Fer-1 ( Figure 2G,H). These results suggested that suppression of PIN1 could induce ferroptosis in CCCs.

### PIN1 regulates ferroptosis via GPX4

**Figure 3:**
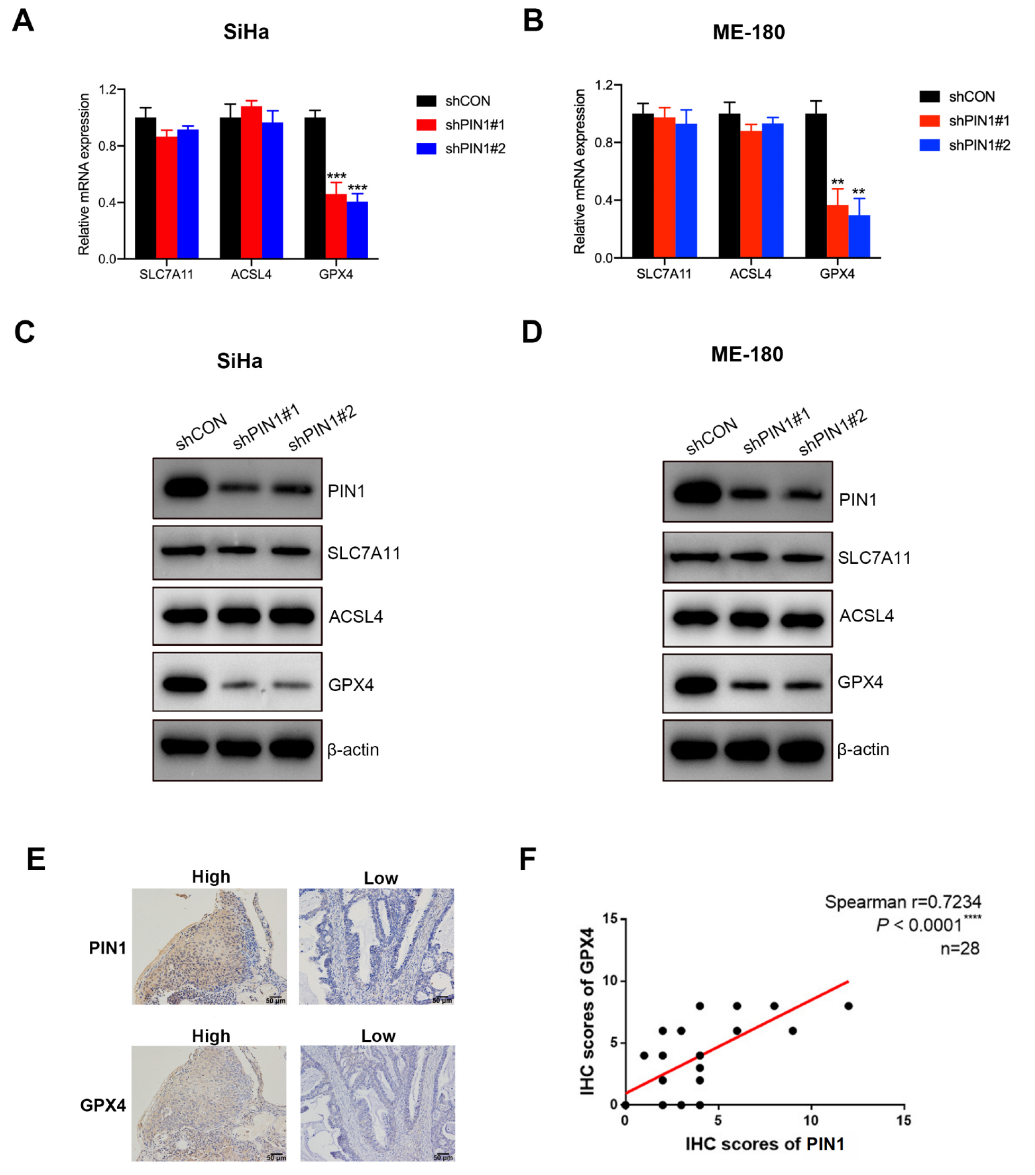
Figure 3 PIN1 regulates ferroptosis via GPX4 (A,B) qRT-PCR was used to evaluate the mRNA levels of SLC7A11, ACSL4 and GPX4 in PIN1-silenced SiHa and ME-180 cells. (C,D) Western blot analysis was used to measure the protein levels of SLC7A11, ACSL4 and GPX4 in PIN1-silenced SiHa and ME-180 cells. (E) Representative images of IHC staining for PIN1 and GPX4 in cervical cancer tissues. Scale bar =50 μm. (F) Spearman correlation analysis of PIN1 expression and GPX4 expression in cervical cancer tissues, as evaluated by the IHC score ( n=28). ** P<0.01, *** P<0.001, and **** P<0.0001.

Ferroptosis is a precise process regulated by a series of key molecules. Fe ^2+^-dependent lipid peroxidation is a key condition for the occurrence of ferroptosis. A previous study showed that acyl-CoA synthetase long-chain family member 4 (ACSL4) could mediate ferroptosis sensitivity by regulating long polyunsaturated ω6 fatty acids on cellular membranes [27]. There is also an antioxidant stress system in cells to resist lipid oxidative damage. Solution carrier family 7 member 11 (SLC7A11) can transport cystine into cells and promote the synthesis of glutathione (GSH), and GSH can increase the enzyme activity of GPX4, remove lipid peroxide and resist lipid peroxidation damage [ 28, 29] . Thus, we determined the mRNA and protein levels of ACSL4, SLC7A11 and GSH. The qPCR and western blot analysis results suggested that the mRNA and protein levels of GPX4, not ACSL4 or SLC7A11, was downregulated in PIN1-knockdown cells ( Figure 3A–D). We also found that PIN1 expression was positively correlated with GPX4 levels in 28 pairs of patient CC samples ( Figure 3E,F). These results showed that PIN1 could regulate ferroptosis via GPX4 and silencing of PIN1 increased the sensitivity of CCCs to RSL3.

### PIN1 regulates the expression of GPX4 via NRF2

**Figure 4:**
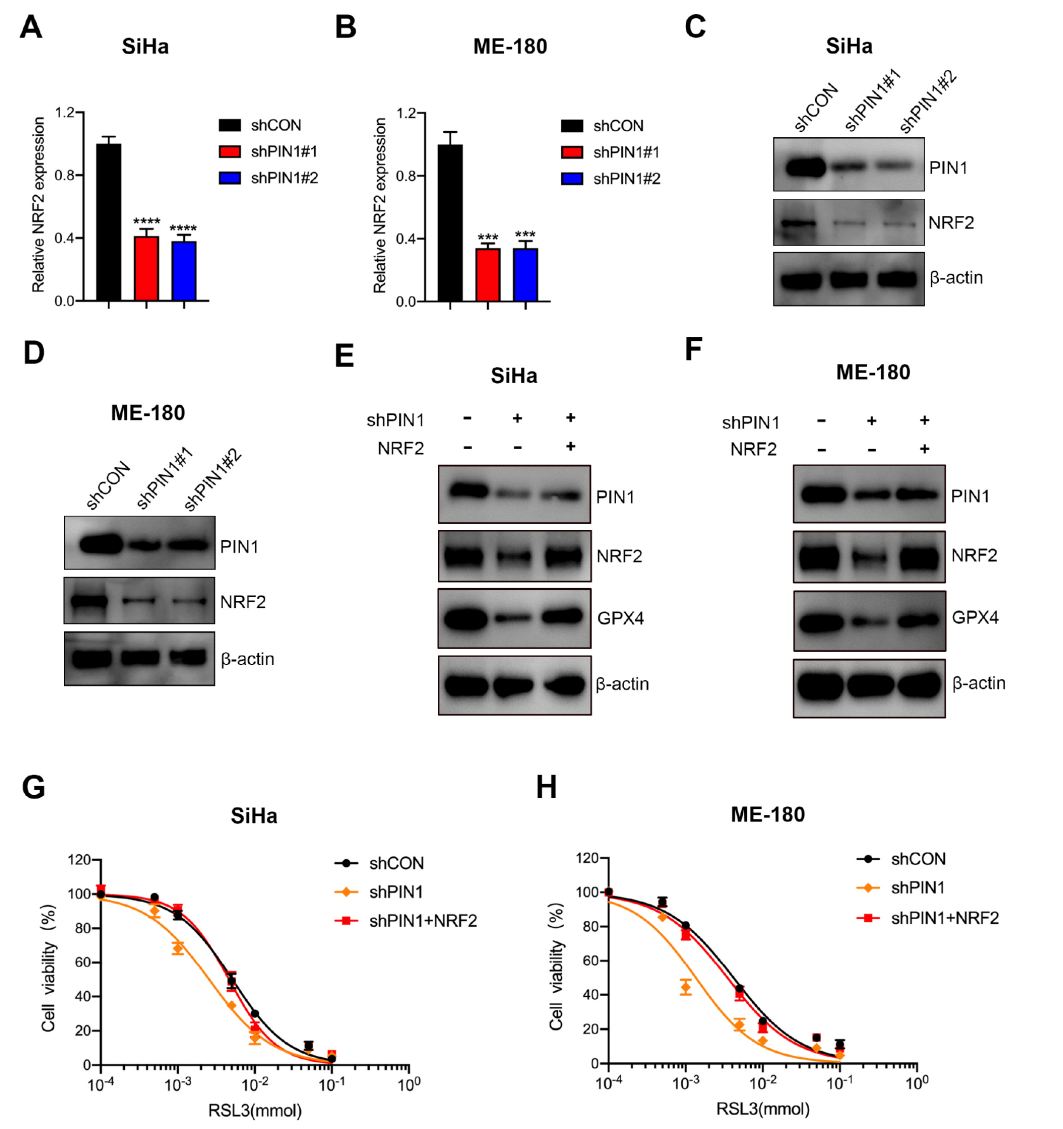
Figure 4 PIN1 regulates GPX4 levels via NRF2 (A,B) The mRNA expression of NRF2 was determined by qRT-PCR in PIN1-silenced SiHa and ME-180 cells. (C,D) The protein expression of NRF2 was determined by western blot analysis in PIN1-silenced SiHa and ME-180 cells. (E,F) The protein expression of GPX4 was determined by western blot analysis in PIN1-silenced and NRF2-overexpressing SiHa and ME-180 cells. (G,H) CCK-8 assay was used to analyze the viability of SiHa and ME-180 cells with downregulated PIN1 and upregulated NRF2 expressions. *** P<0.001, and **** P<0.0001.

NRF2 plays a pivotal role in mediating ferroptosis [30]. Liang *et al*. [31] demonstrated that PIN1 could promote the expression of NRF2 by interacting with c-Myc. In addition, it has been reported that NRF2 can upregulate GPX4 in non-small-cell lung cancer cells [32]. Thus, we hypothesized that PIN1 may mediate the level of GPX4 via NRF2. The qPCR and western blot analysis results indicated that PIN1 knockdown caused a decrease of NRF2 at both the mRNA and protein levels in CCCs ( Figure 4A–D). Moreover, western blot analysis results showed that overexpression of NRF2 reversed the decreased level of GPX4 caused by PIN1 silencing in SiHa and ME-180 cells ( Figure 4E,F). Further results suggested that overexpression of NRF2 attenuated the increased sensitivity to RSL3 induced by PIN1 silencing ( Figure 4G,H). These results demonstrated that PIN1 regulates the expression of GPX4 by mediating NRF2.

### Overexpression of PIN1 suppresses DDP-induced ferroptosis

**Figure 5:**
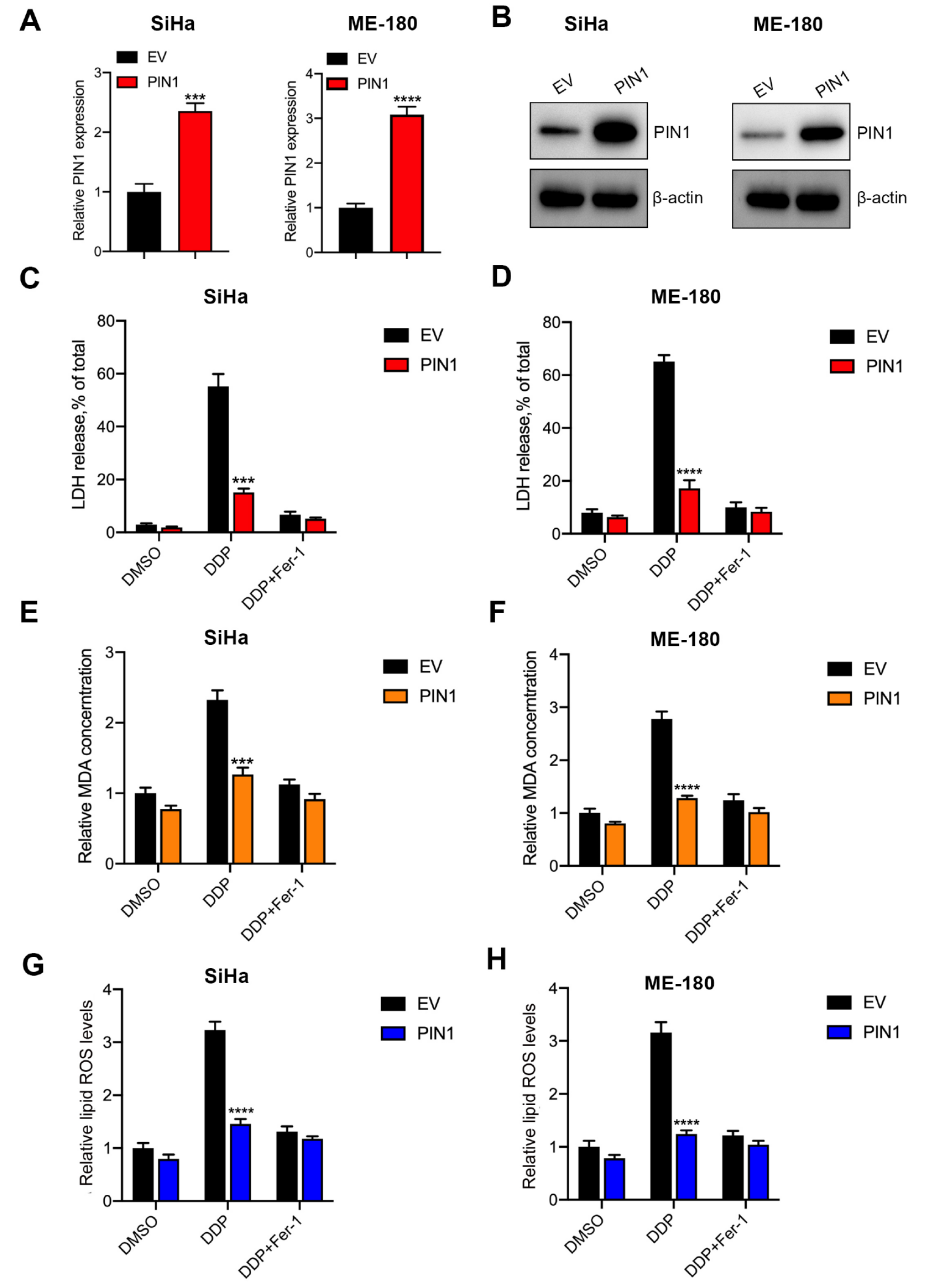
Figure 5 Overexpression of PIN1 suppresses DDP-induced ferroptosis (A) qRT-PCR was conducted to evaluate the overexpression efficiency of PIN1 in SiHa and ME-180 cells. (B) Western blot analysis was used to evaluate the overexpression efficiency of PIN1 in SiHa and ME-180 cells. (C,D) The levels of LDH release were analyzed in PIN1-overexpressing SiHa and ME-180 cells treated with DDP and Fer-1. (E,F) The levels of MDA were analyzed in PIN1-overexpressing SiHa and ME-180 cells treated with DDP and Fer-1. (G,H) The levels of lipid ROS were analyzed in PIN1-overexpressing SiHa and ME-180 cells treated with DDP and Fer-1. *** P<0.001, and **** P<0.0001.

Our results demonstrated that PIN1 could mediate ferroptosis via the NRF2/GPX4 axis in CCCs. It has been shown that DDP promotes ferroptosis by inducing GSH depletion and GPXs inactivation [33]. We hypothesized that PIN1 may regulate the ferroptosis of CCCs induced by DDP. Stable PIN1-overexpressed CCC lines were constructed and the efficiency of overexpression was then confirmed by qPCR and western blot analysis ( Figure 5A,B). The LDH release assay suggested that DDP significantly increased the level of LDH release, which was inhibited by overexpression of PIN1. Additional Fer-1 also suppressed DDP-induced ferroptosis ( Figure 5C,D). Subsequent results showed that upregulation of PIN1 decreased the level of MDA in CCCs treated with DDP, which was further impaired by Fer-1 ( Figure 5E,F). Similarly, the level of lipid ROS in CCCs was increased by DDP, which was inhibited by overexpression of PIN1 or Fer-1 ( Figure 5G,H). Collectively, the ferroptosis induced by DDP was inhibited by overexpression of PIN1.

### PIN1 regulates the sensitivity of CCCs to DDP

**Figure 6:**
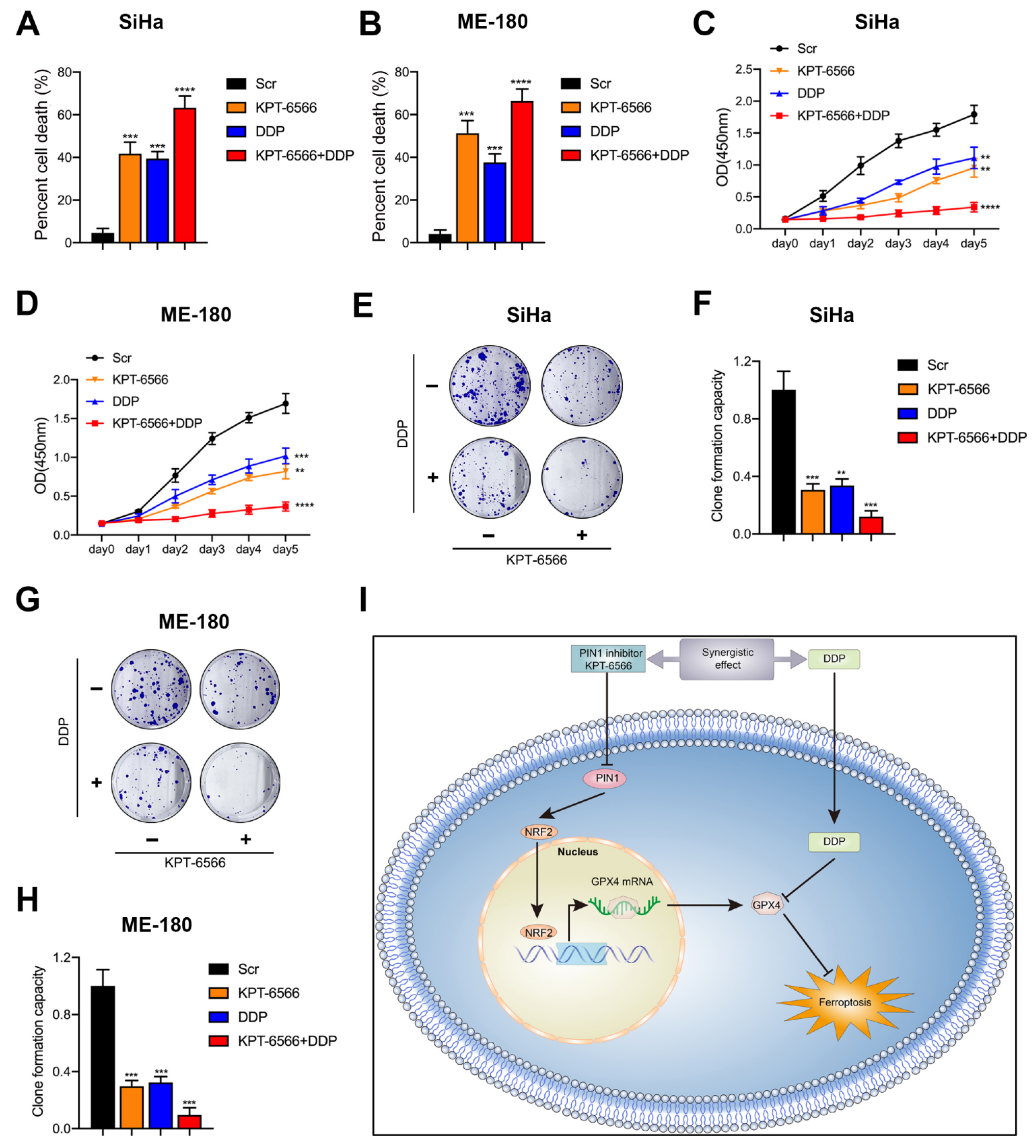
Figure 6 PIN1 regulates DDP sensitivity in CCCs (A,B) CCK-8 assay was used to analyze cell death of SiHa and ME-180 cells treated with DDP and KPT-6566. (C,D) CCK-8 assay was used to evaluate the proliferation of SiHa and ME-180 cells treated with DDP and KPT-6566. (E,F) Colony formation assay was performed to analyze the influence of DDP and KPT-6566 on SiHa cells. (G,H) Colony formation assay was performed to determine the influence of DDP and KPT-6566 on ME-180 cells. ** P<0.01, *** P<0.001, and **** P<0.0001. (I) Schematic representation of the model. The model showed the synergistic effect of DDP and PIN1 inhibitor, KPT-6566, and revealed the underlying mechanism of PIN1 inhibitor-mediated sensitization of DDP by regulating the NRF2/GPX4 axis.

It has been reported that induction of ferroptosis can overcome DDP resistance in head and neck cancer [34]. We sought to investigate the pharmacological significance of inhibiting PIN1. As expected, DDP or KPT-6566, promoted cell death and the lethal effect of DDP combined with KPT-6566 was significantly better than that of DDP or KPT-6566 alone ( Figure 6A,B). CCK-8 assay demonstrated that DDP or KPT-6566 alone inhibited cell growth and the combination of DDP and KPT-6566 further suppressed cell growth ( Figure 6C,D). DDP and KPT-6566 equally inhibited colony formation in CCCs ( Figure 6E–H). The addition of KPT-6566 greatly enhanced the inhibitory effect of DDP ( Figure 6E–H). Therefore KPT-6566 promoted DDP-induced cytotoxicity and the PIN1 inhibitor and DDP may have a synergistic inhibitory effect on CCCs ( Figure 6I).

## Discussion

DDP-based chemotherapy is one of the major treatments for CC; however, subsequent drug resistance often leads to treatment failure. Several studies have reported possible drug resistance mechanisms including apoptosis inhibition, reinforcing DNA repair, enhancing drug efflux and so on [ 8, 35] . However, no valid molecular targets have been identified to overcome drug resistance. Herein, we identified PIN1-mediated ferroptosis as a potential target in chemotherapy-resistant CC. Cell death induced by inhibition of PIN1 was significantly inhibited by the ferroptosis inhibitor rather than the apoptosis or autophagy inhibitor. Moreover, a series of ferroptosis indicators were upregulated by the downregulation of PIN1. Overexpression of PIN1 strongly attenuated DDP-induced ferroptosis. To ascertain the mechanism underlying the impact of PIN1 on chemotherapy sensitivity, we used the PIN1 inhibitor KPT-6566 and found that the PIN1 inhibitor increased sensitivity of cells to DDP via the NRF2/GPX4 axis ( Figure 6I).

PIN1 exerts a crucial impact on cell metabolism, apoptosis and cell proliferation [16]. However, the regulatory role of PIN1 in ferroptosis is still unclear. In the present study, we observed that inhibition of PIN1 significantly promoted cell death, which was efficiently suppressed by Fer-1. However, 3-MA, or Z-VAD-FMK, failed to reverse cell death when PIN1 was suppressed. Suppression of PIN1 may promote ferroptosis more significantly than possible enhancement of apoptosis in CCCs. Silencing of PIN1 also sensitized CCCs to RSL3-induced ferroptosis. Further study suggested that downregulation of PIN1 increased the level of LDH release, MDA concentration, lipid peroxidation and lipid ROS, which were inhibited by Fer-1. From these findings, it is evident that silencing of PIN1 induced ferroptosis.

Furthermore, we determined the expressions of ACSL4, SLC7A11, and GPX4 after PIN1 silencing to investigate the potential mechanism of PIN1-mediated ferroptosis. Interestingly, knockdown of PIN1 downregulated GPX4 at both mRNA and protein levels, but had no impact on the levels of ACSL4 and SLC7A11. Further results suggested that PIN1 expression was positively correlated with GPX4 levels in CC patient tissue samples, indicating that PIN1 mediates ferroptosis by regulating the level of GPX4.

With regard to the underlying mechanism, it was reported that PIN1 is located in the nucleus and interacts with the glycolytic enzyme PGK1 to regulate tumor metabolism [36]. PIN1 can regulate the transcriptional activity of many transcription factors such as c-Myc and CtIP in the nucleus, and then affect the expressions of target genes [37]. NRF2 plays a key role in relieving oxidative stress to maintain redox homeostasis [38]. Furthermore, Abdalkader *et al*. [39] reported that silencing of NRF2 inhibited the expression of GPX4. Therefore, we determined whether NRF2 would affect the regulation of GPX4 by PIN1. The results demonstrated that ferroptosis induced by downregulation of the PIN1/GPX4 axis could be reversed by overexpression of NRF2. The above results revealed that PIN1 may mediate ferroptosis via the NRF2/GPX4 axis. It has been reported that drug-resistant tumor cells are susceptible to ferroptosis [40]. Furthermore, sensitivity to DDP could be meditated by promoting or suppressing ferroptosis [41]. We then examined whether overexpression of PIN1 could regulate the sensitivity of CCCs to DDP. We discovered that upregulation of PIN1 inhibited DDP-induced ferroptosis. Additionally, inhibition of PIN1 increased the sensitivity of CCCs to DDP and PIN1 inhibitor, meanwhile DDP had a synergistic killing effect on CCCs. Similarly, Sun *et al*. [42] reported that the sensitivity of non-small-cell lung cancer cells to DDP could be promoted by FOXO1 and FOXO3a. The effect of DDP could also be significantly enhanced by BIX-01294 pretreatment in nasopharyngeal carcinoma [43]. However, the apoptosis induced by DDP could be resisted by USP31 acetylation in CC [44].

Nevertheless, there are still some limitations in this study. We did not reveal how PIN1 regulates NRF2. The present study demonstrated that PIN1 could regulate NRF2 at the transcriptional level. Liang *et al*. [31] reported that PIN1 transcriptionally activated NRF2 by interacting with c-Myc to bind to the promoter of NRF2 in pancreatic cancer. This regulatory mechanism may also exist in CC. We will further explore the mechanisms in our future studies. Saeidi *et al*. [45] reported that PIN1 could stabilize and constitutively activate NRF2 by competing with Keap1 for Nrf2 binding in breast cancer. However, Kim *et al*. [46] observed that the protein stability of NRF2 was diminished by PIN1 in a ubiquitination-dependent manner [46]. Our present study showed that PIN1 transcriptionally activated NRF2 in CC, which could not rule out the possibility that PIN1 could modulate the stability of NRF2. Further experiments are needed to confirm this possibility. Additionally, *in vivo* experiments were lacking in our study to confirm that silencing of PIN1 can increase the sensitivity of CCCs to DDP. Hence, further *in vivo* study on the role of PIN1-mediated ferroptosis in DDP sensitivity is warranted.

In summary, we revealed the role of PIN1 in ferroptosis and DDP sensitivity in CCCs and preliminarily investigated the underlying mechanism. Our findings showed that PIN1/NRF2/GPX4 may function as potentially therapeutic targets for CC.
